# First person – Liang Zheng

**DOI:** 10.1242/dmm.052407

**Published:** 2025-04-28

## Abstract

First Person is a series of interviews with the first authors of a selection of papers published in Disease Models & Mechanisms, helping researchers promote themselves alongside their papers. Liang Zheng is first author on ‘ Modeling *ANKRD26* 5′-UTR mutation-related thrombocytopenia’, published in DMM. Liang is an assistant professor in the Department of Pathology and Laboratory Medicine, The University of Kansas Medical Center, Kansas City, KS, USA, investigating the molecular mechanisms of thrombosis and hemostasis.


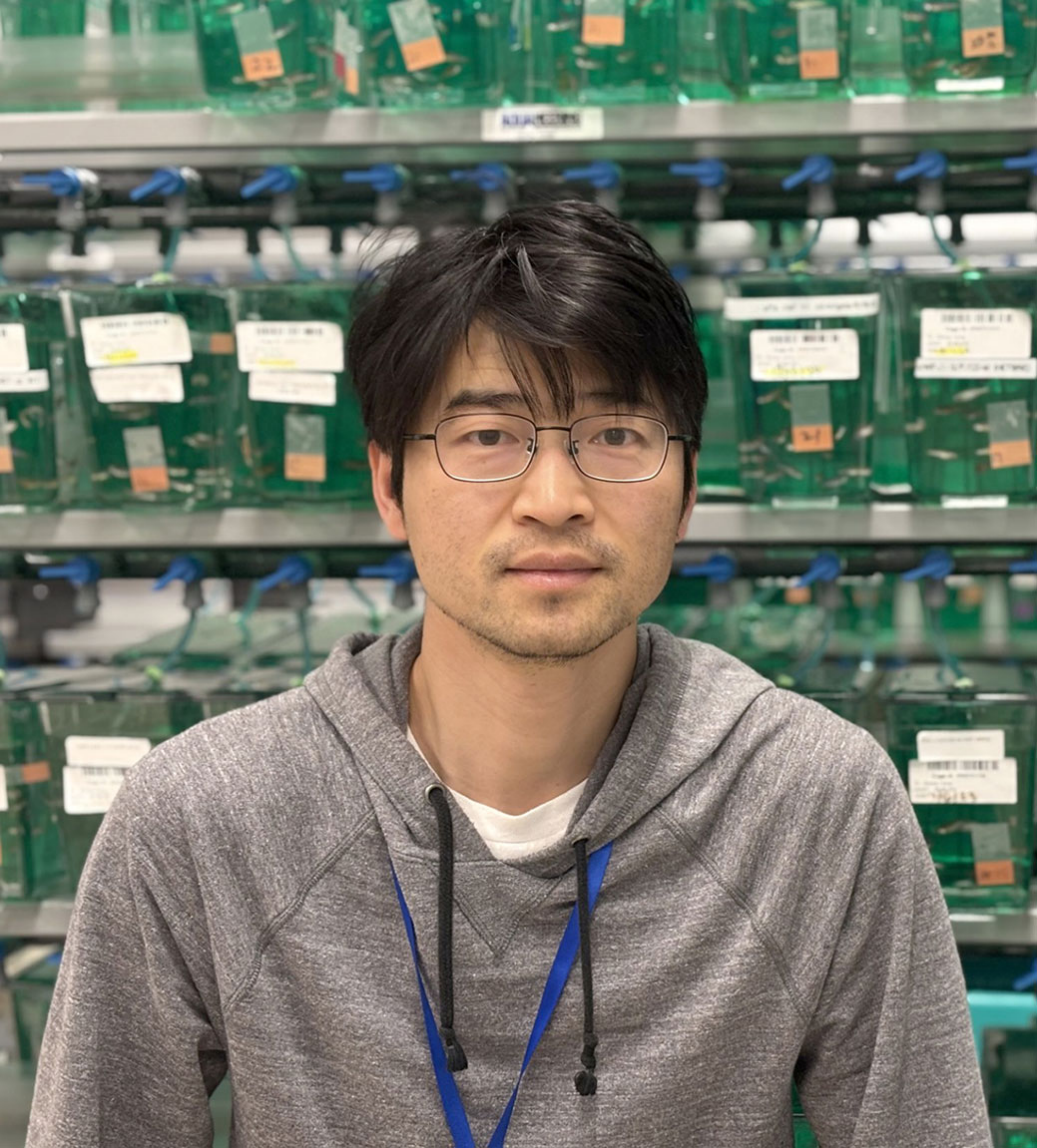


**Liang Zheng**

**Who or what inspired you to become a scientist?**

My fascination with the natural world started in my small hometown, where I grew up surrounded by waterways and a vibrant aquaculture industry. From an early age, I was captivated by the biology of fish, shrimp and crabs. Watching these organisms thrive in their environment sparked my curiosity about how living systems function and adapt. This early interest gradually deepened into a passion for science, leading me to explore the complexities of human health and disease. Today, I study the mechanisms of thrombosis disorders, using both fish and mammalian models to uncover insights that could advance medical research and treatment.

**What is the main question or challenge in disease biology you are addressing in this paper? How did you go about investigating your question or challenge?**

The main question addressed in this paper revolves around understanding the role of *ANKRD26* mutations in thrombocytopenia and hematological malignancies using zebrafish models. The study investigates how mutations in the 5′-untranslated region (5′-UTR) of *ANKRD26*, a gene associated with inherited thrombocytopenia [thrombocytopenia 2 (THC2)], contribute to platelet deficiencies and the potential risk of malignant transformation. The major challenge tackled in this study is determining how *ANKRD26* mutations lead to thrombocytopenia and predispose individuals to hematological malignancies. Understanding this mechanism is crucial for developing targeted therapies for inherited thrombocytopenia and associated blood disorders.

We first established a novel zebrafish model of THC2 by targeting the 5′UTR region of *ankrd26* using CRISPR/Cas9 gene editing. We quantified thrombocytes and analyzed their function in zebrafish with different genotypes. We conducted long-term survival studies and analyzed morphological abnormalities in mutant zebrafish by histological staining and imaging to assess tissue integrity and possible malignant transformation. To explore the molecular mechanisms underlying *ankrd26*-related thrombocytopenia, we performed quantitative proteomic analysis in isolated thrombocytes, identifying potential pathways involved.

**How would you explain the main findings of your paper to non-scientific family and friends?**

This study looked at how changes in a specific gene, *ANKRD26*, affect blood health using tiny fish as a model. We found that, when this gene is altered, the fish had problems with their blood cells, similar to certain human diseases. Some fish even showed signs of more serious health issues over time. To figure out why this happened, we closely examined the fish's blood, body tissues and overall health. We compared fish with the altered gene to normal fish, tracking their survival and any unusual changes. We also studied the tiny building blocks of blood to see how they were different in the affected fish.

Our findings suggest that this gene plays an important role in keeping blood cells working properly. When it doesn't function correctly, it can lead to serious blood disorders. Understanding this could help doctors learn more about similar conditions in people and possibly lead to better treatments in the future.

Our study provides the first *in vivo* evidence that mutations in the 5′-UTR of *ANKRD26* can directly cause spontaneous thrombocytopenia in an animal model.

**What are the potential implications of these results for disease biology and the possible impact on patients?**

Identifying the cause of thrombocytopenia in patients is central to effective clinical management. There is a clear need for better ways to diagnose and treat patients with inherited thrombocytopenia such as THC2, in part to avoid treating patients inappropriately. Our study provides the first *in vivo* evidence that mutations in the 5′-UTR of *ANKRD26* can directly cause spontaneous thrombocytopenia in an animal model. By modeling *ANKRD26*-associated mutations in zebrafish, we reveal how these alterations impact blood cell development and contribute to disease. For individuals with inherited thrombocytopenia, these findings might help doctors provide better guidance on monitoring their condition and managing their risk of complications, including progression to malignancy.

**Figure:**
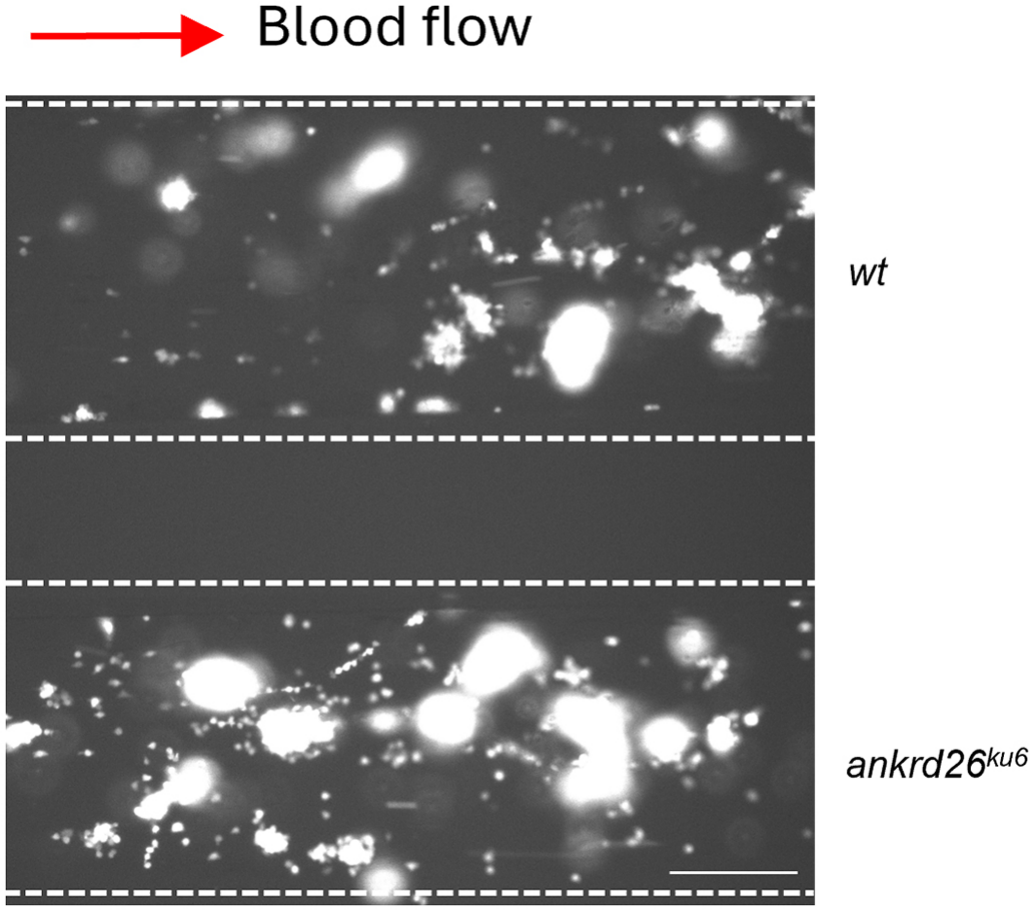
**The surface coverage of fluorescent thrombocytes on a fibrillar collagen-coated surface in a microfluidic channel after perfusion of pooled whole blood obtained from wild-type (wt; top) and *ankrd26^ku6^* (bottom) zebrafish under arterial shear.** Scale bar: 100 µm.

**Why did you choose DMM for your paper?**

We chose DMM due to its strong focus on leveraging model organisms to advance understanding of human disease. DMM is widely recognized for publishing high-quality research that bridges fundamental biological insights with translational and clinical relevance, making it an ideal platform for our work, which employs zebrafish as a model system to investigate the genetic basis of blood disorders. Furthermore, the journal's rigorous peer review process and editorial standards also ensure that the scientific quality and clarity of our work are maintained at the highest level. By publishing in DMM, we aim to reach a specialized audience with a shared interest in disease modeling, while contributing to broader discussions on the pathophysiology and potential therapeutic targets of hematologic diseases.

**Given your current role, what challenges do you face and what changes could improve the professional lives of other scientists in this role?**

As an early-career independent investigator in thrombosis research working with murine and zebrafish models, I face several ongoing challenges. Funding uncertainty and the need to maintain consistent financial support remain constant struggles. Regulatory compliance – particularly managing IACUC protocols, upholding rigorous standards of animal care, and responding to unanticipated protocol inquiries – can be time consuming and administratively burdensome, often diverting focus from active research. Additionally, building and managing a research team while conducting experiments and overseeing daily lab operations can be overwhelming. To better support scientists in similar roles, it is crucial to establish more accessible and sustained funding pathways, including bridge grants and pilot project support. Streamlining compliance processes through standardized animal protocols and increasing institutional assistance with regulatory documentation could substantially reduce administrative burden. Lastly, structured mentorship programs and support for work–life balance are essential for sustaining research careers and mitigating burnout among early-stage investigators.

**What's next for you?**

While advancing our work in the zebrafish model, we have also generated mouse mutants carrying either a loss-of-function *Ankrd26* mutation or a 5′-UTR mutation. Further characterization of these models will help determine whether the mutations lead to defects in megakaryocyte differentiation and platelet production. Additionally, future studies investigating the functional relevance of NINJ1 in *ANKRD26*-associated thrombocytopenia may yield valuable insights into the pathogenesis of not only inherited thrombocytopenia, but also broader hematologic and inflammatory disorders. In terms of my research career, the next steps will focus on solidifying my niche in thrombotic diseases. I aim to prioritize securing sustained National Institutes of Health funding (e.g. an R01), and publishing high-impact studies that bridge fundamental mechanisms with translational relevance. Concurrently, I will continue to build and support my research team, mentor trainees, and contribute to my institution and the broader scientific community through teaching, service and collaboration.

**Tell us something interesting about yourself that wouldn't be on your CV**

I'm a fishing enthusiast. Whether it's the excitement of a big catch or the peaceful solitude of being by the water, fishing is my favorite way to unwind. Plus, I like to think that both fishing and science share the thrill of uncovering something unexpected – whether it's reeling in a fish or making a breakthrough in the lab.
